# 3,4-dimethoxychalcone induces autophagy and reduces neointimal hyperplasia and aortic lesions in mouse models of atherosclerosis

**DOI:** 10.1038/s41419-023-06305-x

**Published:** 2023-11-22

**Authors:** Giulia Cerrato, Carlota Alvarez-Lucena, Allan Sauvat, Yanhua Hu, Sabrina Forveille, Guo Chen, Sylvère Durand, Fanny Aprahamian, Marion Leduc, Omar Motiño, Lisardo Boscá, Qingbo Xu, Oliver Kepp, Guido Kroemer

**Affiliations:** 1 Metabolomics and Cell Biology Platforms, Gustave Roussy Comprehensive Cancer Institute, Villejuif, France; 2 Equipe 11 labellisée Ligue contre le Cancer, Centre de Recherche des Cordeliers, INSERM U1138, Paris, France; 3 Instituto de Investigaciones Biomédicas “Alberto Sols” (CSIC-UAM) and Centro de Investigación en Red en Enfermedades Cardiovasculares (CIBERCV), Madrid, Spain; 4 The Center of Clinical Pharmacology, The Third Xiangya Hospital, Central South University, Changsha, China; 5 Institut du Cancer Paris CARPEM, Department of Biology, Hôpital Européen Georges Pompidou, AP-HP, Paris, France; 6 Present Address: State Key Laboratory of Medicinal Chemical Biology, Frontiers Science Center for Cell Responses, Tianjin Key Laboratory of Protein Science, Haihe Laboratory of Cell Ecosystem, College of Life Sciences, Nankai University, Tianjin, China

**Keywords:** Atherosclerosis, Macroautophagy

## Abstract

Autophagy inducers can prevent cardiovascular aging and age-associated diseases including atherosclerosis. Therefore, we hypothesized that autophagy-inducing compounds that act on atherosclerosis-relevant cells might have a protective role in the development of atherosclerosis. Here we identified 3,4-dimethoxychalcone (3,4-DC) as an inducer of autophagy in several cell lines from endothelial, myocardial and myeloid/macrophagic origin, as demonstrated by the aggregation of the autophagosome marker GFP-LC3 in the cytoplasm of cells, as well as the downregulation of its nuclear pool indicative of autophagic flux. In this respect, 3,4-DC showed a broader autophagy-inducing activity than another chalcone (4,4- dimethoxychalcone), spermidine and triethylene tetramine. Thus, we characterized the potential antiatherogenic activity of 3,4-DC in two different mouse models, namely, (i) neointima formation with smooth muscle expansion of vein segments grafted to the carotid artery and (ii) genetically predisposed *ApoE*^−/−^ mice fed an atherogenic diet. In the vein graft model, local application of 3,4-DC was able to maintain the lumen of vessels and to reduce neointima lesions. In the diet-induced model, intraperitoneal injections of 3,4-DC significantly reduced the number of atherosclerotic lesions in the aorta. In conclusion, 3,4-DC stands out as an autophagy inducer with potent antiatherogenic activity.

## Introduction

Macroautophagy (to which we refer to as ‘autophagy’) is an evolutionarily conserved cellular process culminating in the lysosomal degradation of dispensable and potentially harmful material present in the cytoplasm of cells [1]. As such, autophagy facilitates the cellular adaptation to multiple stresses including nutrient starvation, oxidative stress, hypoxia or infection [2]. Importantly, autophagy is also required for controlling systemic metabolism, and immune responses, as well as for the maintenance of organismal homeostasis [3–6]. With age, autophagy declines in most organs including the components of the cardiovascular system, and disabled autophagy is thought to be one of the major hallmarks of systemic and cardiovascular aging [7–9].

In the past, we showed that autophagy inducers can prevent or mitigate cardiovascular diseases, including myocardium infarction and heart failure [9–14]. Due to their galenic properties and reduced cost, small molecules are particularly interesting for the prevention or treatment of cardiovascular diseases. Thus, high nutritional spermidine uptake is associated with reduced cardiovascular morbidity and mortality in humans [15] and spermidine supplementation reduces the severity of atherosclerosis in mice [16]. Spermidine acts against normal cardiac aging, as well as against high-salt diet-induced cardiac insufficiency [17, 18]. The copper-chelating agent triethylenetetramine (TETA) improves cardiovascular function and can induce the regression of pressure overload-induced cardiac hypertrophy [19, 20]. Another autophagy inducer, 4,4’-dimethoxychalcone (4,4’-DC) prevents myocardial necrosis after ligation of the left coronary artery [21]. Furthermore, another, structurally related chalcone, 3,4-dimethoxychalcone (3,4-DC), prevents myocardial necrosis [22] and induces autophagy in multiple mouse organs [22].

Atherosclerosis is the most prevalent aging-associated cardiovascular disease, providing the pathogenic substratum of most cases of myocardial infarction, stroke, aortic aneurysm and arterial occlusion affecting internal organs or the femoral artery. The etiology of atherosclerosis appears complex but involves an important dysfunction of innate and cognate immune effectors [23], with macrophage-mediated inflammatory responses [24] and the formation of foam cells (macrophages exhibiting the accumulation of lipid droplets in their cytoplasm) [25] as prominent elements of the disease process. Given the anti-inflammatory effects of autophagy [11, 26] and the important anti-atherosclerotic role of lipophagy (a subtype of autophagy causing the removal of lipid droplets) [27], we wondered whether the administration of pharmacological autophagy inducers might protect against the development of atherosclerosis.

Based on these premises, we attempted to identify the best strategy to prevent atherosclerosis by searching for agents among the aforementioned compounds that would induce autophagy in all cardiovascular disease-relevant cell types, i.e., cardiomyocytes, endothelial cells and macrophages. As we report here, 3,4-DC stood out as a broad autophagy inducer. In a series of in vivo experiments involving two distinct mouse models of atherosclerosis, we obtained preclinical evidence indicating that 3,4-DC can efficiently prevent this condition.

## Results

### 3,4-DC induces autophagy in cardiovascular disease-relevant cell lines in vitro

**Fig. 1:**
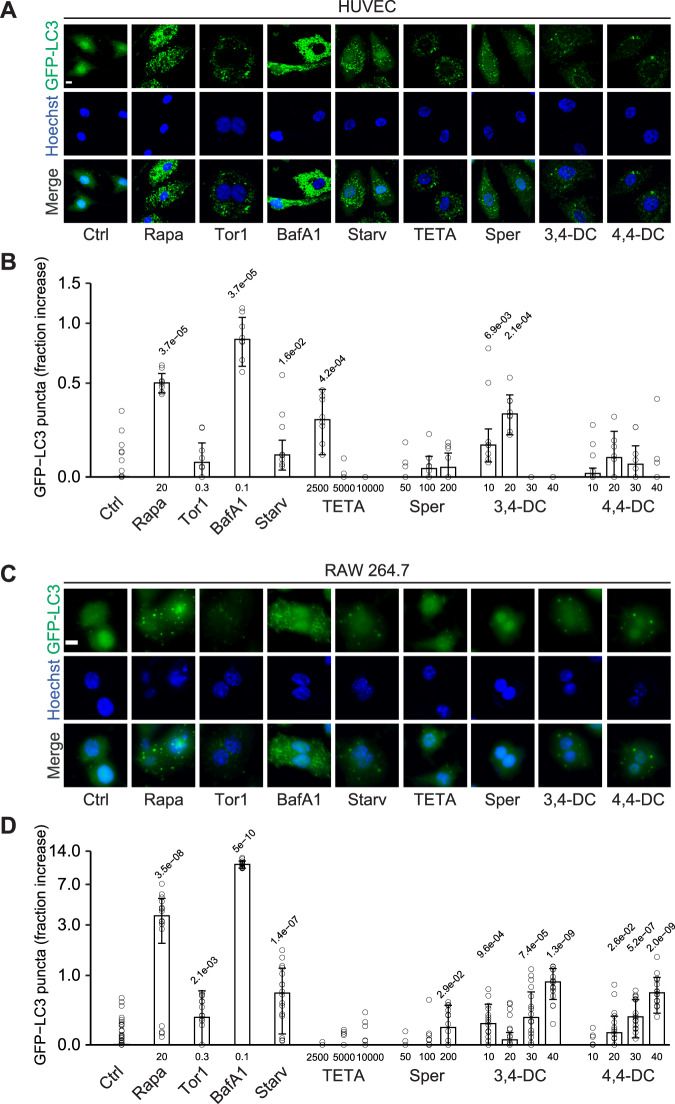
Autophagy induction by caloric restriction mimetics in endothelial cells and cardiomyoblasts. Human vascular endothelial HUVEC cells (**A**, **B**) and murine RAW 264.7 macrophages (**C**, **D**) stably expressing GFP-LC3 were treated with the indicated agents at the indicated concentrations (µM) for 6 hours. After fixation and staining with Hoechst 33342, images were acquired and the formation of GFP-LC3 puncta was assessed by confocal microscopy. Representative images are depicted in (**A**, **C**) (size bar equals 10 µm) and the relative average number of GFP-LC3 positive puncta is shown as bar charts using a pseudo-logarithmic scale (**B**, **D**). Data are represented as median ± median absolute deviation (MAD) and significance was tested by means of a Mann–Whitney U-test. P-values are indicated. Rapamycin (Rapa), torin 1 (Tor1), bafilomycin A1 (BafA1), serum deprivation/starvation (Starv), triethylenetetramine (TETA), spermidine (Sper), 3,4-dimetoxychalcone (3,4-DC), 4,4-dimetoxychalcone (4,4-DC).

**Fig. 2:**
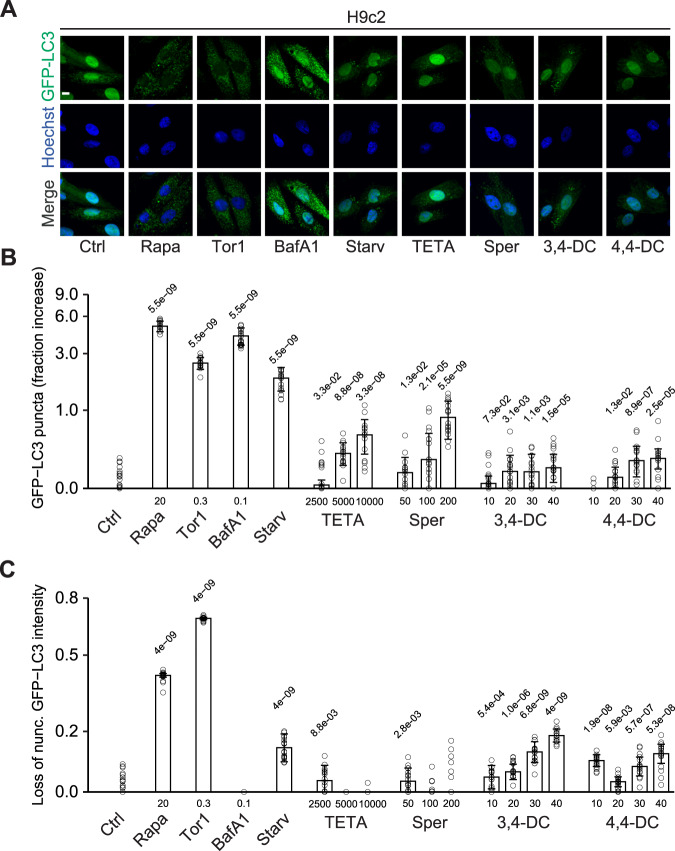
Autophagy induction by caloric restriction mimetics in cardiomyoblast H9c2 cells. Rat cardiomyoblast H9c2 cells stably expressing GFP-LC3 were treated with the indicated agents at the indicated concentrations (µM) for 6 hours. After fixation and staining with Hoechst 33342, images were acquired and the formation of GFP-LC3 puncta as well as the cytoplasmic translocation of GFP-LC3 were assessed by confocal microscopy. Representative images are depicted in (**A**). Size bar equals 10 µm. The average number of GFP-LC3 positive puncta was enumerated (**B**) and the cytoplasmic translocation of GFP-LC3 was calculated as loss of nuclear fluorescent signal (**C**). Both were normalized to control and are shown as bar charts using a pseudo-logarithmic scale (**B**, **C**). Data are represented as median ± MAD and significance was tested by means of a Mann–Whitney U-test. *P*-values are indicated. Rapamycin (Rapa), torin 1 (Tor1), bafilomycin A1 (BafA1), serum deprivation/starvation (Starv), triethylenetetramine (TETA), spermidine (Sper), 3,4-dimetoxychalcone (3,4-DC), 4,4-dimetoxychalcone (4,4-DC).

**Fig. 3:**
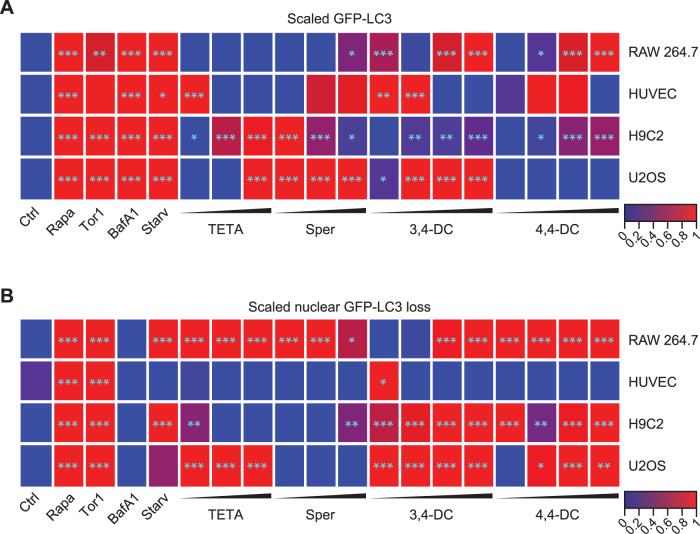
Autophagy induction by caloric restriction mimetics. Human vascular endothelial HUVEC cells, murine RAW 264.7 macrophages, rat cardiomyoblast H9c2 and human osteosarcoma U2OS cells stably expressing GFP-LC3 were treated with the indicated agents at the indicated concentrations for 6 h. GFP-LC3 puncta formation (**A**) and nuclear fluorescence intensity loss (**B**) are reported as medians in a heatmap after sigmoid smoothing. *P*-values were categorized and plotted as follows: *p < 0.05; **p < 0.01; ***p < 0.001. Rapamycin (Rapa), torin 1 (Tor1) bafilomycin A1 (BafA1), serum deprivation/starvation (Starv), triethylenetetramine (TETA), spermidine (Sper), 3,4-dimetoxychalcone (3,4-DC), 4,4-dimetoxychalcone (4,4-DC).

When added to human vascular endothelium HUVEC cells (Fig. 1A, B), murine RAW 264.7 macrophages (Fig. 1C, D), rat cardiomyoblasts H9c2 cells (Fig. 2A, B), or human osteosarcoma U2OS cells (Fig. S1), stably expressing green fluorescent protein (GFP) fused with microtubule-associated protein 1 light chain 3 alpha/beta MAP1LC3A/B (best known as LC3), 3,4-dimethoxychalcone (3,4-DC) significantly induced the aggregation of LC3 across cell lines in cytoplasmic dots. These effects occurred in a dose-dependent fashion. Other tested agents such as triethylenetetramine (TETA), spermidine and 4,4’-DC depicted less consistent and partially cell type-specific effects. Autophagic flux inducers such as rapamycin, torin1 and serum starvation as well as the lysosomal acidification inhibitor bafilomycin A1 were used as positive controls that reproducibly induced GFP-LC3 dot formation across cell lines (Figs. 1–2, Fig. S1). We also quantified the abundance of nuclear LC3 that decreases with autophagic flux as a result of LC3 deacetylation and its subsequent nuclear export [28]. 3,4-DC reduced the intensity of the GFP-LC3-dependent fluorescence in nuclei stained with Hoechst 33342 in all 4 cell types (Figs. 2C, 3B, Fig. S1C, S2).

In conclusion, when compared to the three other candidate agents (4,4’-DC, spermidine, TETA), 3,4-DC appeared to mediate the broadest pro-autophagic effects in the 4 different cell lines (Fig. 3A, B). Therefore, we decided to investigate 3,4-DC in further detail.

### Pharmacokinetics and pharmacodynamics of 3,4-DC

**Fig. 4:**
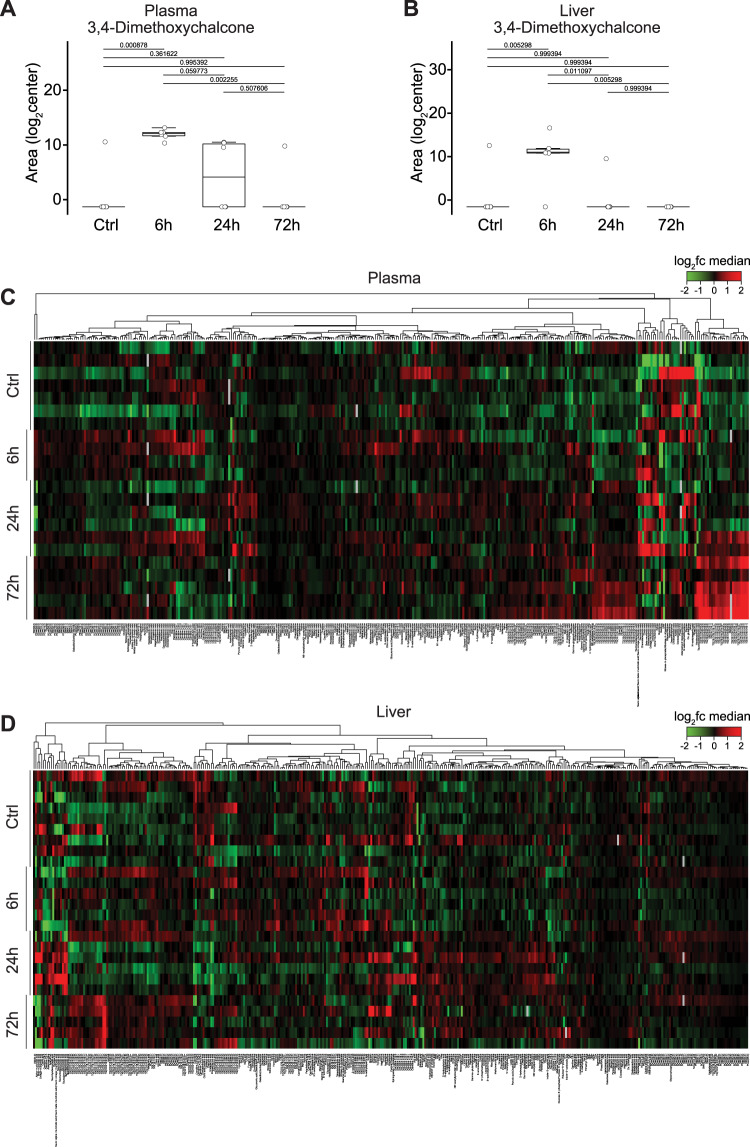
Metabolomic assessment of plasma and liver from 3,4-DC treated mice. Mice were treated with 230 mg/kg 3,4-dimetoxychalcone (3,4-DC) intraperitoneally and samples were prepared 6, 24 and 72 h after treatment and subjected to mass spectrometric analysis. Untargeted analysis revealed the bioavailability of 3,4-DC 6 and 24 h of treatment in plasma and liver. Data are depicted as boxplots with ANOVA post hoc tuckey test with FDR adjustment in (**A**, **B**). Targeted analysis depicted a change in triglycerides towards later timepoints. Data are depicted as heatmaps in (**C**, **D**). Data are log2 normalized and centered around the average abundance computed for each metabolite. Red/green colors are ion signal higher/lower than average and missing values are displayed as grey. Samples are organized in a supervised way. Metabolites are clustered following the ward.D2 algorithm, with Euclidean distance.

We previously showed that intraperitoneal injection of 3,4-DC (230 mg/kg) induced autophagic flux (measured at 24 h) in the liver and the heart. This effect occurs well beyond the clearance of 3,4-DC that is bioavailable in plasma and liver 6 hours after injection but is barely detectable at 24 h in the liver, as determined by mass spectrometry (Fig. 4A, B). We also measured the effects of 3,4-DC on whole-body metabolism using mass spectrometric metabolomics. Of note, although 3,4-DC became undetectable at 72 h after injection in plasma and liver, it did have metabolic effects that lasted for 72 h, consisting in an increase of hepatic and circulating triglycerides (Fig. 4C, D; Fig. S3, S4).

In sum, 3,4-DC is bioavailable, though endowed with a relatively short half-life, and it mediates autophagy-inducing and metabolic effects that can be detected in vivo, in laboratory mice.

### 3,4-DC inhibits neointimal hyperplasia in vein graft model

**Fig. 5:**
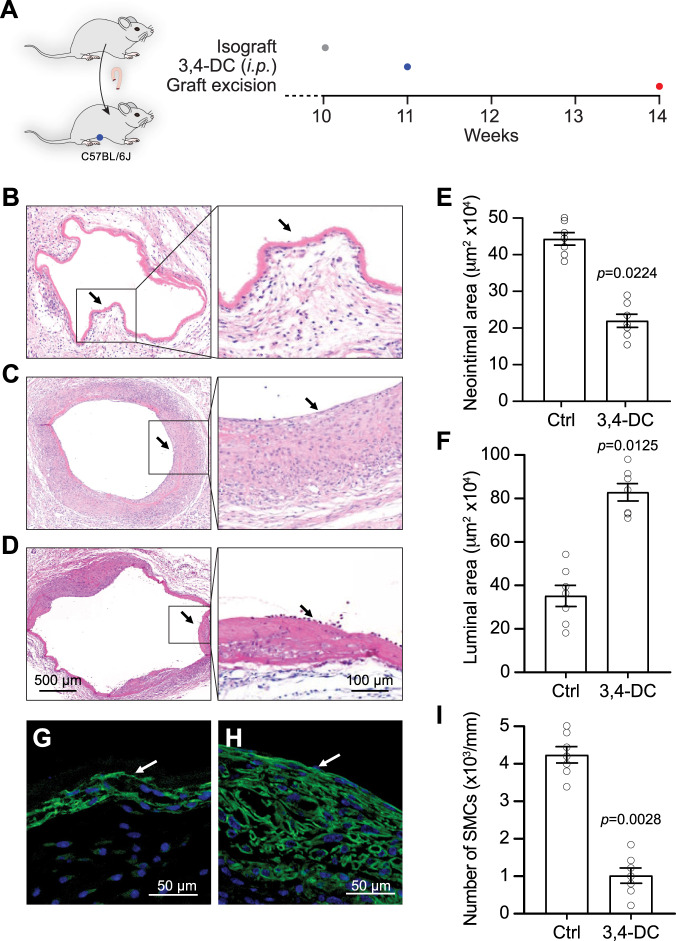
Morphological changes and quantification of neointimal lesions in vein allografts. (**A**) A schematic overview of the experimental protocol is shown. (**B**) Representative images depict sections of vein segments from mice before grafting, or 4 weeks after isografting them into the carotid artery of mice and local treatment with vehicle (**C**) or 3,4-dimetoxychalcone (3,4-DC) (**D**). Tissue sections were stained with hematoxylin and eosin (**B**–**D**). Arrows indicate the endothelium in representative images. The luminal and neointimal areas were quantified by morphometry (**E**, **F**). The sections from vehicle-only treated (**G**) or 3,4-DC treated (**H**) mice were subjected to immunofluorescence staining with antibodies specific for the smooth muscle cell antigen α−actin-FITC (green) and counterstained with DAPI (blue; nuclei). Positive cells in the neointimal lesions were counted (**I**). Quantitative data indicate values for individual mice (*n* = 7). Data are represented as mean values ± SEM and significance was tested by Student’s t test.

Vein grafts develop neointimal lesion and atherosclerosis in the presence of hyperlipidemia [29], which was used to validate the effectiveness of the reagent on lesion formation (Fig. 5A). Normal murine veins contain few layers of cells surrounded by adventitial connective tissue (Fig. 5B). In contrast grafts of veins to carotid arteries of mice exhibited marked alterations 4 weeks after the procedure. In particular, grafted veins displayed neointimal hyperplasia, i.e., thickening of the vessel wall by multiple cell layers, as well as increased smooth muscle cell and matrix protein accumulation (Fig. 5C). Treatment of the vein grafts with vehicle (pluronic-127 gel) alone did not significantly influence neointima formation. However, vein grafts from 3,4-DC-treated mice showed markedly reduced neointima lesions 3 weeks after 3,4-DC injection (Fig. 5D). Parallel immunofluorescence staining with antibodies against LC3B showing the formation of autophagic puncta, validated the induction of autophagy caused by 3,4-DC in vivo (Fig S5). To statistically analyze vein graft lesions, we quantified the luminal and neointimal areas. 3,4-DC treatment (3 weeks, *n* = 7) maintained the lumen of the vessel (Fig. 5E) and reduced neointimal thickness by 50% to 70 % compared to vehicle-treated controls (Fig. 5F). Immunofluorescence staining using monoclonal antibodies against α-actin demonstrated the presence of abundant smooth muscle cells (SMCs) in venous bypass graft lesions 4 weeks after surgery (Fig. 5G, H). The number of positive-stained SMCs was markedly reduced in 3,4-DC-treated vein grafts at 4 weeks (Fig. 5I).

Altogether, these data indicate that 3,4-DC can to prevent signs of allotransplantation-induced vascular transplantation such as neointimal hyperplasia due to the proliferation of SMCs leading to progressive obstruction of the lumen.

### 3,4-DC inhibits atherosclerosis in *ApoE*^−/−^ mice on a high-fat diet

**Fig. 6:**
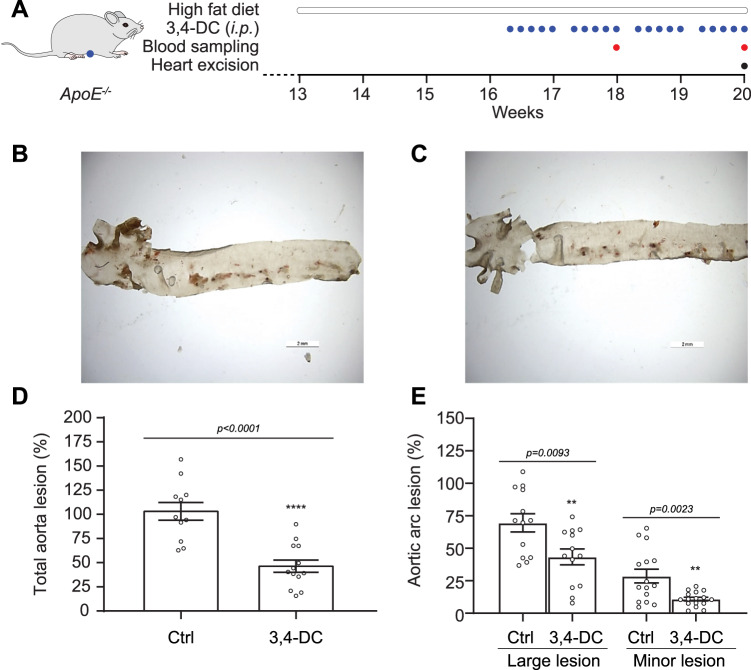
3,4-DC reduces the accumulation of fats in aortic arch and thoracic aorta in *Apoe*^*−/−*^ mice fed high-fat diet (HFD). **A** Twelve-weeks-old *Apoe*^*−/−*^ mice were feed with HFD for 4 weeks in the presence of 3,4-dimetoxychalcone (3,4-DC) or vehicle (Ctrl). Then atherosclerotic lesions were quantified by histochemistry with oil red O. **B**, **C** Representative images of atheroma plaque from *Apoe*^*−/−*^ mice treated with vehicle or 3,4-DC for quantification Size bar equals 2 mm. **D**, **E** Microscopical quantification of atheroma plaque in the total aortic surface of *Apoe*^*−/−*^ mice treated with vehicle (*n* = 13) or 3,4-DC (*n* = 13). Data are represented as mean ± SEM and significance was tested by Student’s t test.

Next, we took advantage of a standard rodent model of atherosclerosis consisting of the administration of a high-fat diet to *ApoE*^−/−^ mice [30]. Three-month-old male *ApoE*^−/−^ mice were fed an atherogenic high-fat diet (HFD) for one month. During this period the animals received 3,4-DC 5 times per week (230 mg/kg in corn oil) or vehicle alone (Fig. 6A). Of note, examination of plasma lipids and lipoproteins did not reveal any long-term shifts induced by 3,4-DC (Fig. S6). Nonetheless, 3,4-DC did attenuate the severity of aortic atherosclerosis detected by oil red staining and stereomicroscopic quantification (Fig. 6B, C). The reduction of the number of lesions applied to the total aorta (Fig. 6D), as well as to the aortic arc in which both large and minor lesions were less frequent in 3,4-DC-treated mice than in vehicle-treated controls (Fig. 6E).

In conclusion, 3,4-DC reduces vascular injury in a second mouse model of atherosclerosis induced by HFD in *ApoE*^−/−^ mice.

### Concluding remarks

Here we showed that the caloric restriction mimetic (CRM) 3,4-DC is a potent inducer of autophagy in cardiovascular disease-relevant cell types, it is bioavailable after intraperitoneal injection (as well as upon topical application) and can be used for the prevention of atherosclerosis in two rather different mouse models, (i) neointima formation in vein segments grafted to the carotid artery and (ii) genetically predisposed *ApoE*^−/−^ mice fed an atherogenic diet. Furthermore, we showed that 3,4-dimethoxychalcone induces autophagy in multiple different cell lines and that it hence might be useful for the treatment of the downstream consequences of arteriosclerosis (such as myocardial infarction or stroke).

3,4-DC was identified as a stimulator of autophagic flux when a library of 200 polyphenols and polyamines were screened for their capacity to induce the formation of cytoplasmic GFP-LC3 dots in U2OS cells [22, 31] and then validated as an inducer of autophagy in vivo, in the heart, liver [22] and spinal cord [32]. In these organs, 3,4-DC potently activates pro-autophagy transcription factors including transcription factor EB (TFEB)- and E3 (TFE3) [22, 32]. As with other CRMs, 3,4-DC was found to mediate broad health-improving effects. Thus, 3,4-DC protected the hearts from mice against coronary artery ligation-induced necrosis. This effect depended on autophagy induction in cardiomyocytes, because conditional knockout of the essential autophagy gene *Atg7* abolished the cardioprotective effect of 3,4-DC [22]. In addition, 3,4-DC improved the anticancer immune response in the context of immunogenic chemotherapies, and this effect was lost upon genetic inactivation of autophagy in malignant cells [22]. Moreover, 3,4-DC favored functional recovery after spinal cord injury, and this effect is lost upon inactivation of TFEB [32]. Of note, topically applied 3,4-DC also turned out to mediate beneficial effects against ultraviolet-A-induced skin damage [33]. Although this effect was attributed to the antioxidant activity of 3,4-DC, autophagy has previously been implicated in the natural and drug-induced protection of the skin against ultraviolet insult and photoaging [34–36]. Hence, it is possible, yet remains to be demonstrated that the skin-protective effects of 3,4-DC are secondary to local autophagy induction.

It is noteworthy that a single injection of 3,4-DC enhanced triglyceride levels in the liver and in the blood, but that a longer, repeated exposure to 3,4-DC failed to durably alter triglycerides after 15 days (and 10 injections) or 4 weeks (and 20 injections). Moreover, long-term treatment with 3,4-DC did not alter the plasma concentrations of cholesterol, free fatty acids, low-density lipoprotein (LDL) and high-density lipoprotein (HDL). These findings suggest that 3,4-DC mediates its antiatherogenic activity by a direct action on blood vessels rather than by effects on systemic metabolism. To demonstrate such a direct effect, it would be necessary to conditionally knockout essential autophagy genes in relevant cell types (e.g., endothelial cells and macrophages) and to demonstrate that 3,4-DC loses its atheroprotective action. These experiments are now being planned in our laboratory.

## Material and methods

### Cell culture and treatment

Chemicals were purchased from Sigma Aldrich (St Louis, MO, USA), torin 1 was received from Tocris Bioscience (Bristol, UK) and 3,4-DC as well as 4,4-DC were bought from ABCR (Karlsruhe, Germany). Culture media came from Gibco-Thermofisher Scientific (Carlsbad, CA, USA). Human osteosarcoma U2OS cells, rat cardiomyoblast H9c2 cells, human vascular endothelium HUVEC cells and murine RAW 264.7 macrophages were obtained from the American Type Culture Collection (ATCC) and cultured under standard cell culture conditions according to furnisher’s recommendations (5% CO_2_; 37 °C in a water-saturated atmosphere within a cell culture incubator (HeraCell, Heraeus, Germany)). Cells were stably transduced with lentiviral particles coding for green fluorescent protein (GFP)-microtubule-associated protein 1 light chain 3 alpha/beta MAP1LC3A/B (also known as LC3) (Lentibrite, EMD Millipore, Burlington, MA, US). Subsequently, stably expressing cells were selected by means of a FACS DIVA (Becton Dickinson, Franklin Lakes, NJ, USA).

### Automated image acquisition and analysis

Cells stably expressing GFP-LC3 were seeded in μClear imaging plates (Greiner Bio-One) and let adhere for 24 h. The next day, cells were treated for 6 h, then fixed with 3.7% formaldehyde containing 1 µg/mL Hoechst 33342 for 1 h at room temperature before acquisition employing a robot-assisted automated confocal fluorescence bioimager IXM-C bioimager (Molecular Devices, Sunnyvale, CA, USA) equipped with an Aura II light source (Lumencor, Beaverton, OR, USA), standard excitation and emission filters (Semrock, Rochester, NY, USA), a 16-bit monochrome sCMOS Andor Zyla camera (Belfast, Northern Ireland) and a 20X PlanAPO objective (Nikon, Tokyo, Japan). At least four view fields per well were acquired and then images were segmented and analyzed with R (https://www.r-project.org), using the *EBImage and RBioFormats* packages available from the Bioconductor repository (https://www.bioconductor.org), the *MetaxpR* package (https://github.com/kroemerlab/MetaxpR), as well as the *MorphR* package (https://github.com/kroemerlab/MorphR). Exclusively viable cells were subjected to further analysis. The primary region of interest (ROI) was defined by a polygon mask around the nucleus allowing for the enumeration of cells, the detection of morphological alterations of the nucleus and the quantification of GFP-LC3. Secondary cytoplasmic ROIs were used for the quantification of GFP-LC3 puncta, a segmentation mask of high-intensity dots was generated in the cytoplasm of cells. After the exclusion of cellular debris and dead cells, GFP-LC3 nuclear intensity loss and the number of GFP-LC3 dots were computed as the fractional increase of untreated controls, statistically evaluated by means of a Mann–Whitney U-test and graphically depicted using R software using a pseudo-logarithmic scale with a sigma of 0.25. For plotting heatmaps, both GFP-LC3 puncta and nuclear GFP-LC3 loss were normalized to positive controls and obtained values were smoothened using sigmoidal transformation.

### In vivo bioavailability

C57BL/6 mice were purchased from Janvier Labs (Le Genest-Saint-Isle, France). Preclinical evaluation was conducted in compliance with the EU Directive 63/2010 and protocols 2019_030_20590 and were approved by the Ethical Committee of the Gustave Roussy Campus Cancer (CEEA IRCIV/IGR no. 26, registered at the French Ministry of Research). Mice were housed in a temperature-controlled environment with 12 h light/dark cycles and received food and water ad libitum. For exploration mice were injected intraperitoneally with a single dose of 230 mg/kg 3,4-DC, dissolved in corn oil. At the end of the experiment, the animals were anesthetized by isoflurane inhalation. Liver tissue was extracted and plasma was obtained from whole blood by centrifugation at 200 g for 10 min at 4 °C and kept at −80 °C prior to extraction.

### Determination of metabolic changes in mice liver and plasma

For sample preparation 30 mg tissue were solubilized in 1 mL cold lysis buffer (methanol, H_2_O, chloroform; −20 °C) in polypropylene microcentrifuge tubes with ceramic beads and then homogenized three times for 20 s at 5500 rpm using a Precellys 24 tissue homogenizer (Bertin Technologies, Montigny-le-Bretonneux, France), followed by centrifugation (10 min at 15,000 g; 4 °C). Then the upper phase was separated and 270 µL were used for gas chromatography coupled to mass spectrometry (GC/MS) and 250 µL were used for ultra-high-pressure liquid chromatography coupled to mass spectrometry (UHPLC/MS). Metabolomic analysis was conducted as described before [37]. Statistical tests of significance were performed with Kruskall-Wallis and Dunn’s post hoc test with Benjamini-Hochberg adjustment regarding the targeted data, and ANOVA post hoc tuckey test with FDR adjustement regarding the untargeted data. Hierarchical metabolite clustering was performed using the MetaboAnalyst 3.0 suite71 with log2-normalized data.

### Vein graft procedure

All animal experiments and protocols were approved by the institutional committee of the department of laboratory animals, Central South University; approval reference No. 2021/159. C57BL/6 J were purchased from Hunan SJA Laboratory Animal Co., Ltd (Hunan, China). The mice were maintained on a light/dark (12/12 h) cycle at 22 °C receiving food and water ad libitum. The vein graft procedure was similar to that described previously [38, 39]. Briefly, 3-month-old mice were anesthetized, and a vein segment (vena cava) was grafted between the two ends of the carotid artery. Immediately after vessel grafting, 50 µl of 3,4-DC (60 µM) in 20% pluronic-127 gel (pH 7.2) were applied to the adventitia. On contact with the tissues, the solutions gelled immediately, generating a translucent layer that enveloped the grafted vessel segment. One week after grafting, mice were treated with 230 mg/kg 3,4-DC dissolved in corn oil (Sigma-Aldrich) or an equivalent volume of vehicle alone by intraperitoneal injection.

### Histology and lesion quantification

Mice were anesthetized and perfused with 0.9% NaCl solution via cardiac puncture in the left ventricle and subsequently, perfusion fixed with 4% phosphate-buffered formaldehyde (pH 7.2) for 2 and 5 min, respectively as described previously [39]. The grafts were harvested at 4 weeks postoperatively, processed by routine histology and embedded in paraffin. Sections (5 µm) from the center of the graft were stained with hematoxylin and eosin (HE) for histological evaluation [39]. The procedure used for lesion quantification was the same as previously described [38].

### Immunohistochemical staining

Prior to incubation with the antibody antigen retrieval was performed by chymotrypsin digestion (500 mg/L chymotrypsin 500 mg/L CaCl_2_ in PBS) for 30 min. Immunohistochemical staining was conducted with a FITC-conjugated monoclonal anti-smooth muscle α-actin antibody (Sigma-Aldrich). For autophagy detection, vein graft sections were stained with an antibody specific to LC3 II (Sigma-Aldrich) overnight at 4 °C, then incubated with Alexa-Fluor secondary antibody (Invitrogen) for 1 h at room temperature and nuclei were counterstained with DAPI. LC3-positive cells were enumerated against the total number of cells in the section.

### *ApoE*^*-/-*^ model of atherosclerosis

Animal studies were approved by the local ethics committee, and all animal procedures were in agreement with the EU Directive 2010/63 and Recommendation 2007/526/EC, enforced by the Spanish law under the Real Decreto 53/2013 (PROEX number 42.8/23). *Apoe*^−/−^ mice [40, 41] were obtained from Charles River (JAX mice stock #002052,). Only male mice were used for the experiments because these animals are prone to develop atherogenesis, as previously described [30, 42].

All mice were genotyped by polymerase chain reaction (PCR) from ear samples using standard procedures. In order to accelerate the development of atherosclerotic lesions, at 12 weeks of age, males were placed on high-fat diet (HFD, 10.7% total fat, 0.75% cholesterol; Sniff, Soest, Germany) for 4 weeks. The experimental group was treated five times a week during 4 weeks with 230 mg/kg of 3,4-DC [22] dissolved in corn oil (Sigma-Aldrich) used as vehicle. The control group was treated five times a week during 4 weeks with an equivalent volume of vehicle alone. After this feeding period, mice were anesthetized by isoflurane (2-5%) for blood collection from the orbital sinus and plasma was obtained by centrifugation at 2000 g for 10 min at 4 °C. After blood collection, mice were euthanized by CO_2_ inhalation.

### Lesion quantification

After cardiac perfusion with PBS supplemented with 5 mM of EDTA, hearts were harvested and fixed in 4% paraformaldehyde (PFA) for 24 h at 4 °C, washed twice with PBS and conserved at 4 °C. Aortas were whole-mount stained with 0.2% oil red O (ORO, Sigma) in 80% methanol and opened longitudinally to expose the luminal surface. Images were acquired using a Leica MZ6 SZX10 stereomicroscope (Leica Microsystems, Wetzlar, Germany) coupled to a Leica DFC300 digital camera (Leica Microsystems). The planimetric area of atherosclerotic plaques was measured in millimeters using ImageJ. To avoid specific bias due to potential differences in lesion shape, cross-sections of the entire lesion were analyzed and averaged.

### Quantification and statistical analysis of in vivo data

All values are expressed as means ± SEM. Statistical calculations were performed using GraphPad Prism 9 (GraphPad Software Inc.; San Diego, CA, USA). After calculating for normality by D’Agostino–Pearson omnibus test, either a non-parametric test (Mann–Whitney U-test), or a normality test (unpaired Student’s t test with Welch’s correction) was used as appropriate. Statistical significance was deemed at *p* values < 0.05. Removal of outliers was assessed by the ROUT method. Statistical tests and p values are specified for each panel in the respective figure legend. As indicated in the figure legends n refers to the number of individual animals for in vivo and ex vivo assays.

### Plasma lipid and lipoprotein analysis

Plasma concentrations of total cholesterol, free cholesterol, LDL-cholesterol, HDL-cholesterol, and triglycerides. The serum content was measured using a Dimension RxL Max Integrated Chemistry System (Siemens Healthineers, Erlangen, Germany). All analyses were performed by specialized staff at the CNIC Animal Facility (Madrid, Spain). Whole blood was collected from the orbital sinus and plasma was obtained by centrifugation at 2000 g for 10 min at 4 °C. Plasma concentrations of total cholesterol, free cholesterol, LDL-cholesterol, HDL-cholesterol, and triglycerides were measured. Serum content was measured using a Dimension RxL Max Integrated Chemistry System (Siemens Healthineers, Erlangen, Germany). All analyses were performed by specialized staff at the CNIC Animal Facility.

### Supplementary information

Supplemental files

## Data Availability

Metabolomic data generated here is available on Mendeley data (10.17632/fd48ys2m2k.1). Additional data and material can be shared upon reasonable request.

## References

[1] Galluzzi L, Bravo-San Pedro JM, Demaria S, Formenti SC, Kroemer G (2017). Activating autophagy to potentiate immunogenic chemotherapy and radiation therapy. Nat Rev Clin Oncol.

[2] Galluzzi L, Vitale I, Aaronson SA, Abrams JM, Adam D, Agostinis P (2018). Molecular mechanisms of cell death: recommendations of the Nomenclature Committee on Cell Death 2018. Cell Death Differ.

[3] Galluzzi L, Pietrocola F, Bravo-San Pedro JM, Amaravadi RK, Baehrecke EH, Cecconi F (2015). Autophagy in malignant transformation and cancer progression. EMBO J.

[4] Galluzzi L, Pietrocola F, Levine B, Kroemer G (2014). Metabolic control of autophagy. Cell.

[5] Rubinsztein DC, Marino G, Kroemer G (2011). Autophagy and aging. Cell.

[6] Ma Y, Galluzzi L, Zitvogel L, Kroemer G (2013). Autophagy and cellular immune responses. Immunity.

[7] Aman Y, Schmauck-Medina T, Hansen M, Morimoto RI, Simon AK, Bjedov I (2021). Autophagy in healthy aging and disease. Nat Aging.

[8] Lopez-Otin C, Blasco MA, Partridge L, Serrano M, Kroemer G (2023). Hallmarks of aging: An expanding universe. Cell.

[9] Abdellatif M, Rainer PP, Sedej S, Kroemer G (2023). Hallmarks of cardiovascular ageing. Nat Rev Cardiol.

[10] Bravo-San Pedro JM, Kroemer G, Galluzzi L (2017). Autophagy and Mitophagy in Cardiovascular Disease. Circ Res.

[11] Levine B, Kroemer G (2019). Biological Functions of Autophagy Genes: A Disease Perspective. Cell.

[12] Abdellatif M, Sedej S, Carmona-Gutierrez D, Madeo F, Kroemer G (2018). Autophagy in Cardiovascular Aging. Circ Res.

[13] Motino O, Lambertucci F, Anagnostopoulos G, Li S, Nah J, Castoldi F (2022). ACBP/DBI protein neutralization confers autophagy-dependent organ protection through inhibition of cell loss, inflammation, and fibrosis. Proc Natl Acad Sci USA.

[14] Montegut L, Joseph A, Chen H, Abdellatif M, Ruckenstuhl C, Motino O (2023). High plasma concentrations of acyl-coenzyme A binding protein (ACBP) predispose to cardiovascular disease: Evidence for a phylogenetically conserved proaging function of ACBP. Aging Cell.

[15] Kiechl S, Pechlaner R, Willeit P, Notdurfter M, Paulweber B, Willeit K (2018). Higher spermidine intake is linked to lower mortality: a prospective population-based study. Am J Clin Nutr.

[16] Michiels CF, Kurdi A, Timmermans JP, De Meyer GRY, Martinet W (2016). Spermidine reduces lipid accumulation and necrotic core formation in atherosclerotic plaques via induction of autophagy. Atherosclerosis.

[17] Eisenberg T, Abdellatif M, Schroeder S, Primessnig U, Stekovic S, Pendl T (2016). Cardioprotection and lifespan extension by the natural polyamine spermidine. Nat Med.

[18] Eisenberg T, Abdellatif M, Zimmermann A, Schroeder S, Pendl T, Harger A (2017). Dietary spermidine for lowering high blood pressure. Autophagy.

[19] Liu J, Chen C, Liu Y, Sun X, Ding X, Qiu L (2018). Trientine selectively delivers copper to the heart and suppresses pressure overload-induced cardiac hypertrophy in rats. Exp Biol Med (Maywood).

[20] Pietrocola F, Castoldi F, Madeo F, Kroemer G (2020). Triethylenetetramine (trientine): a caloric restriction mimetic with a new mode of action. Autophagy.

[21] Carmona-Gutierrez D, Zimmermann A, Kainz K, Pietrocola F, Chen G, Maglioni S (2019). The flavonoid 4,4’-dimethoxychalcone promotes autophagy-dependent longevity across species. Nat Commun.

[22] Chen G, Xie W, Nah J, Sauvat A, Liu P, Pietrocola F (2019). 3,4-Dimethoxychalcone induces autophagy through activation of the transcription factors TFE3 and TFEB. EMBO Mol Med.

[23] Fernandez DM, Giannarelli C (2022). Immune cell profiling in atherosclerosis: role in research and precision medicine. Nat Rev Cardiol.

[24] Soehnlein O, Libby P (2021). Targeting inflammation in atherosclerosis - from experimental insights to the clinic. Nat Rev Drug Discov.

[25] Back M, Yurdagul A, Tabas I, Oorni K, Kovanen PT (2019). Inflammation and its resolution in atherosclerosis: mediators and therapeutic opportunities. Nat Rev Cardiol.

[26] Schroder K, Tschopp J (2010). The inflammasomes. Cell.

[27] Laval T, Ouimet M (2023). A role for lipophagy in atherosclerosis. Nat Rev Cardiol.

[28] Huang R, Xu Y, Wan W, Shou X, Qian J, You Z (2015). Deacetylation of nuclear LC3 drives autophagy initiation under starvation. Mol Cell.

[29] Ni Z, Lyu L, Gong H, Du L, Wen Z, Jiang H (2023). Multilineage commitment of Sca-1(+) cells in reshaping vein grafts. Theranostics.

[30] Gonzalez-Ramos S, Fernandez-Garcia V, Recalde M, Rodriguez C, Martinez-Gonzalez J, Andres V (2020). Deletion or Inhibition of NOD1 Favors Plaque Stability and Attenuates Atherothrombosis in Advanced Atherogenesis. Cells.

[31] Kabeya Y, Mizushima N, Ueno T, Yamamoto A, Kirisako T, Noda T (2000). LC3, a mammalian homologue of yeast Apg8p, is localized in autophagosome membranes after processing. EMBO J.

[32] Zhang H, Ni W, Yu G, Geng Y, Lou J, Qi J (2023). 3,4-Dimethoxychalcone, a caloric restriction mimetic, enhances TFEB-mediated autophagy and alleviates pyroptosis and necroptosis after spinal cord injury. Theranostics.

[33] Fatmasari E, Zulkarnain AK, Kuswahyuning R (2021). 3,4-dimethoxychalcone novel ultraviolet-A-protection factor in conventional sunscreen cream. J Adv Pharm Technol Res.

[34] Qiang L, Sample A, Shea CR, Soltani K, Macleod KF, He YY (2017). Autophagy gene ATG7 regulates ultraviolet radiation-induced inflammation and skin tumorigenesis. Autophagy.

[35] Wang M, Charareh P, Lei X, Zhong JL (2019). Autophagy: Multiple Mechanisms to Protect Skin from Ultraviolet Radiation-Driven Photoaging. Oxid Med Cell Longev.

[36] Umar SA, Shahid NH, Nazir LA, Tanveer MA, Divya G, Archoo S (2021). Pharmacological Activation of Autophagy Restores Cellular Homeostasis in Ultraviolet-(B)-Induced Skin Photodamage. Front Oncol.

[37] Viltard M, Durand S, Perez-Lanzon M, Aprahamian F, Lefevre D, Leroy C (2019). The metabolomic signature of extreme longevity: naked mole rats versus mice. Aging (Albany NY).

[38] Dietrich H, Hu Y, Zou Y, Dirnhofer S, Kleindienst R, Wick G (2000). Mouse model of transplant arteriosclerosis: role of intercellular adhesion molecule-1. Arterioscler Thromb Vasc Biol.

[39] Zou Y, Dietrich H, Hu Y, Metzler B, Wick G, Xu Q (1998). Mouse model of venous bypass graft arteriosclerosis. Am J Pathol.

[40] Zhang SH, Reddick RL, Piedrahita JA, Maeda N (1992). Spontaneous hypercholesterolemia and arterial lesions in mice lacking apolipoprotein E. Science.

[41] Plump AS, Smith JD, Hayek T, Aalto-Setala K, Walsh A, Verstuyft JG (1992). Severe hypercholesterolemia and atherosclerosis in apolipoprotein E-deficient mice created by homologous recombination in ES cells. Cell.

[42] Gonzalez-Ramos S, Paz-Garcia M, Rius C, Del Monte-Monge A, Rodriguez C, Fernandez-Garcia V (2019). Endothelial NOD1 directs myeloid cell recruitment in atherosclerosis through VCAM-1. FASEB J.

