# Supplementary material for: 3,4-dimethoxychalcone induces autophagy and reduces neointimal hyperplasia and aortic lesions in mouse models of atherosclerosis
Source: Cell Death Dis. 2023 Nov 22;14(11):758. doi: 10.1038/s41419-023-06305-x (PMC10663525; doi:10.1038/s41419-023-06305-x)
Supplement: Supplementary file 1 — Supplemental files [file 41419_2023_6305_MOESM1_ESM.pdf]

Figure S1. Autophagy induction by caloric restriction mimetics in epithelial U2OS cells. Human osteosarcoma U2OS cells stably expressing GFP-LC3 were treated with the indicated agents at the indicated concentrations for 6 hours. After fixation and staining with Hoechst 33342, images were acquired and the formation of GFP-LC3 puncta as well as the cytoplasmic translocation of nuclear GFP-LC3 were assessed by confocal microscopy. Representative images are depicted in (**A**). Size bar equals 10 µm. Numerical values were statistically evaluated, normalized to control and are shown as bar-charts in (**B,C**) using a pseudo-logarithmic scale. For the assessment of autophagy, the average number of GFP-LC3 positive dots per cell was quantified and is expressed as fractional increase of untreated control, while cytoplasmic translocation of GFP-LC3 was calculated as loss of nuclear fluorescent signal. Data are represented as median ± MAD and significance were tested by means of a Mann–Whitney U-test. P-values are indicated. Rapamycin (Rapa), torin-1 (Tor1), bafilomycin A1 (BafA1), serum deprivation/starvation (Starv), triethylenetetramine (TETA), spermidine (Sper), 3,4-dimetoxychalcone (3,4-DC), 4,4-dimetoxychalcone (4,4-DC).

Figure S2. Nuclear cytoplasmic GFP-LC3 translocation induced by caloric restriction mimetics in HUVEC cells and RAW macrophages. Human vascular endothelial HUVEC cells and murine RAW 264.7 macrophages stably expressing GFP-LC3 were treated with the indicated agents at the indicated concentrations for 6 hours. After fixation and staining with Hoechst 33342, images were acquired, the cytoplasmic translocation of nuclear GFP-LC3 was assessed by confocal microscopy and calculated as loss of nuclear fluorescent signal. Both were normalized to control and are shown as bar charts using a pseudo-logarithmic scale (**A,B**). Data are represented as median ± MAD and significance were tested by means of a Mann–Whitney U-test. P-values are indicated. Rapamycin (Rapa), torin-1 (Tor1), bafilomycin A1 (BafA1),

serum deprivation/starvation (Starv), triethylenetetramine (TETA), spermidine (Sper), 3,4-dimetoxychalcone (3,4-DC), 4,4-dimetoxychalcone (4,4-DC).

Figure S3. Mass spectrometric assessment of triglycerides in plasma and liver from 3,4-DC treated mice. Mice were treated with 230 mg/kg 3,4-dimethoxychalcone (3,4-DC) intraperitoneally, samples were prepared at the indicated timepoints and then subjected to mass spectrometric analysis. Data are depicted as heatmaps and resemble an excerpt of the larger metabolomic assessment depicted in Figure 4. Fatty acids annotated with ** have been controlled with available standards.

Figure S4. Statistical assessment of triglycerides in plasma and liver from 3,4-DC treated mice. Heatmaps show plasma (**A**) and liver (**B**) metabolites, as fold change of the mean. Test of significance was conducted with a Kruskal Wallis and Dunn's post-hoc test with Benjamini-Hochberg adjustment, and results are depicted as asterisks (*$p < 0.05$; **$p < 0.01$ and ***$p < 0.005$).

Figure S5. LC3 puncta formation in neointimal lesions of vein grafts. (**A**) Vein segments were grafted into the carotid artery and locally treated with vehicle (Ctrl) or 3,4-dimethoxychalcone (3,4-DC) and harvested 4 weeks after grafting. The sections were subjected to immunofluorescence staining with anti-LC3 antibody (red) and DAPI (blue). Size bars equal 10 µm. Positive cells in the neointimal lesions were enumerated and data is summarized as bar chart in (**B**). Data are represented as mean ± SEM of the indicated number of repeats (n=6). Data were statistically evaluated by means of a Mann–Whitney U-test. P-value is indicated.

Figure S6. **Representation of plasmatic parameters determination in *Apoe*$^{-/-}$ mice treated with vehicle (n = 15) or with 3,4-DC (n = 15) at day 15 and 30 after treatment and HFD.** Twelve-week-old *Apoe*$^{-/-}$ mice were feed with HFD for 4 weeks in the presence of 3,4-dimetoxychalcone (3,4-DC) or vehicle (Ctrl). (**A**) Free cholesterol (mg/dL), (**B**) total cholesterol (mg/dL), (**C**) pancreatic Lipase (U/L), (**D**) High-density lipoprotein (HDL) (mg/L), (**E**) low-density lipoprotein (LDL) (mg/dL), (**F**) triglycerides (mg/dL) and (**G**) non-esterified fatty acids (NEFA) (mmol/L) were determined. Data are represented as mean ± SEM of the indicated number (n) of repeats. Data were statistically evaluated by Student's t test.

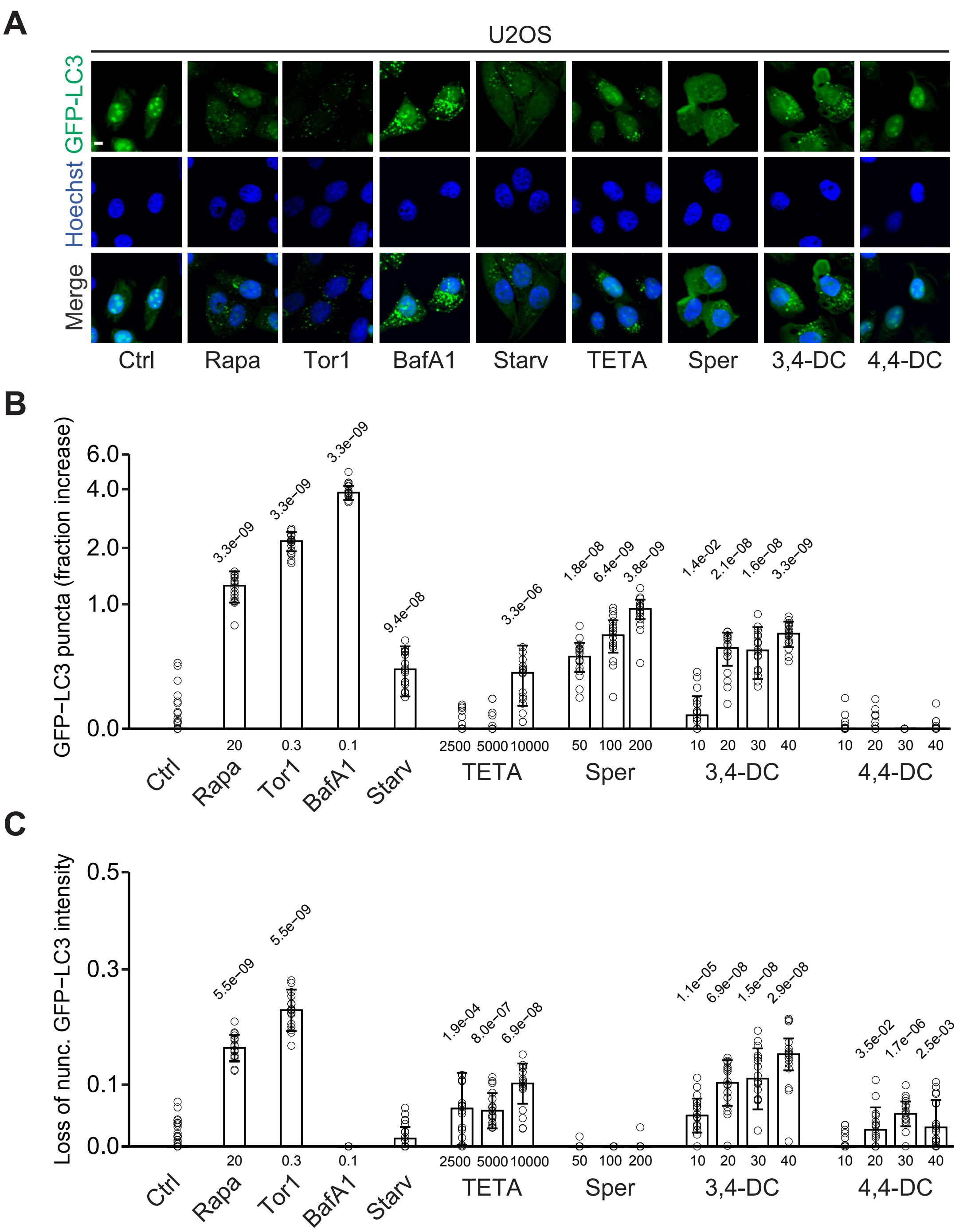
A
U2OS
GFP-LC3
Hoechst
Merge
Ctrl
Rapa
Tor1
BafA1
Starv
TETA
Sper
3,4-DC
4,4-DC
B
GFP-LC3 puncta (fraction increase)
6.0
4.0
2.0
1.0
0.0
3.3e-09
3.3e-09
3.3e-09
9.4e-08
3.3e-06
1.8e-08
6.4e-09
3.8e-09
1.4e-02
2.1e-08
1.6e-08
3.3e-09
20
0.3
0.1
2500
5000
10000
50
100
200
10
20
30
40
10
20
30
40
Ctrl
Rapa
Tor1
BafA1
Starv
TETA
Sper
3,4-DC
4,4-DC
C
Loss of nunc. GFP-LC3 intensity
0.5
0.3
0.1
0.0
5.5e-09
5.5e-09
1.9e-04
8.0e-07
6.9e-08
1.1e-05
6.9e-08
1.5e-08
2.9e-08
3.5e-02
1.7e-06
2.5e-03
20
0.3
0.1
2500
5000
10000
50
100
200
10
20
30
40
10
20
30
40
Ctrl
Rapa
Tor1
BafA1
Starv
TETA
Sper
3,4-DC
4,4-DC

Figure S1

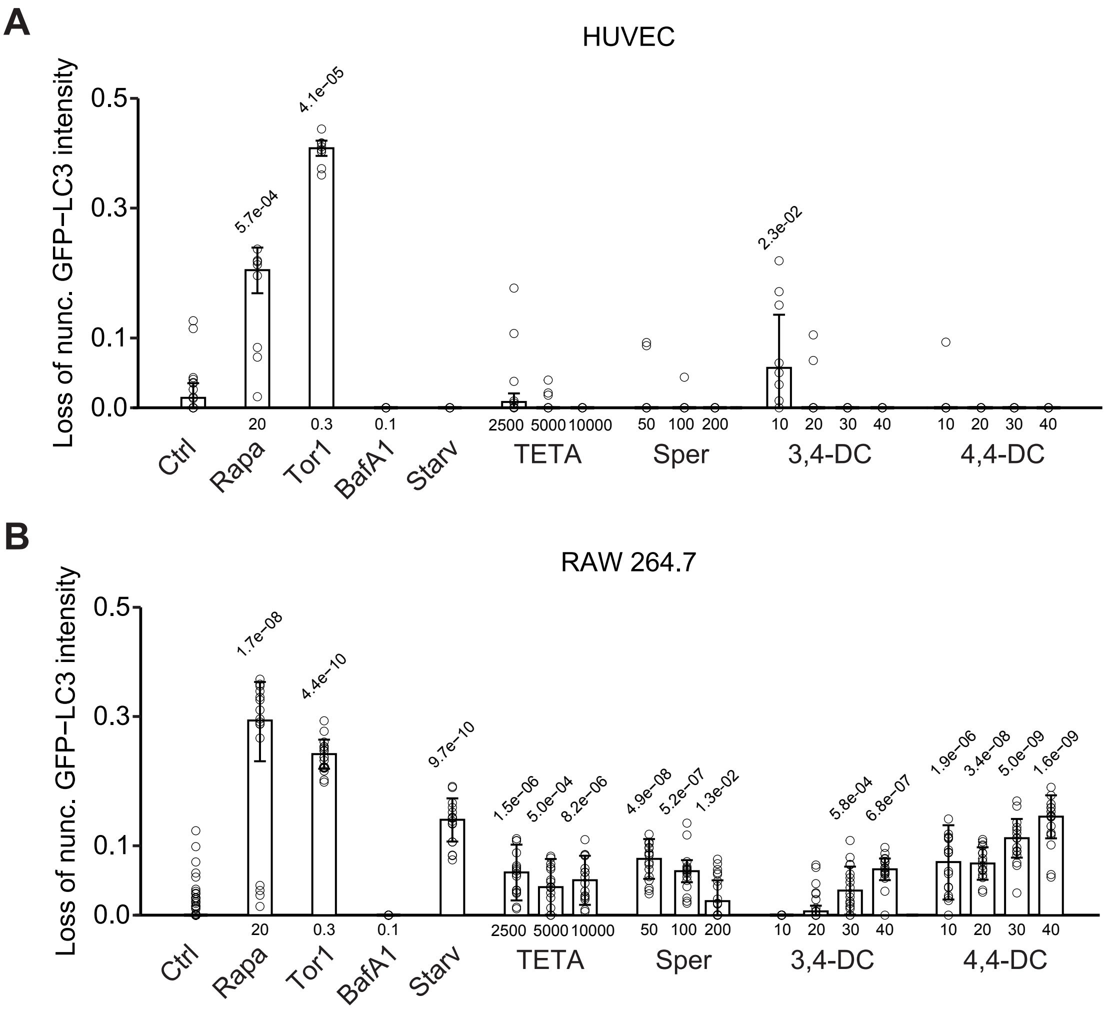
A
HUVEC
Loss of nunc. GFP−LC3 intensity
0.0
0.1
0.3
0.5
5.7e−04
4.1e−05
2.3e−02
20
0.3
0.1
2500
5000
10000
50
100
200
10
20
30
40
10
20
30
40
Ctrl
Rapa
Tor1
BafA1
Starv
TETA
Sper
3,4-DC
4,4-DC
B
RAW 264.7
Loss of nunc. GFP−LC3 intensity
0.0
0.1
0.3
0.5
1.7e−08
4.4e−10
9.7e−10
1.5e−06
5.0e−04
8.2e−06
4.9e−08
5.2e−07
1.3e−02
5.8e−04
6.8e−07
1.9e−06
3.4e−08
5.0e−09
1.6e−09
20
0.3
0.1
2500
5000
10000
50
100
200
10
20
30
40
10
20
30
40
Ctrl
Rapa
Tor1
BafA1
Starv
TETA
Sper
3,4-DC
4,4-DC

Figure S2

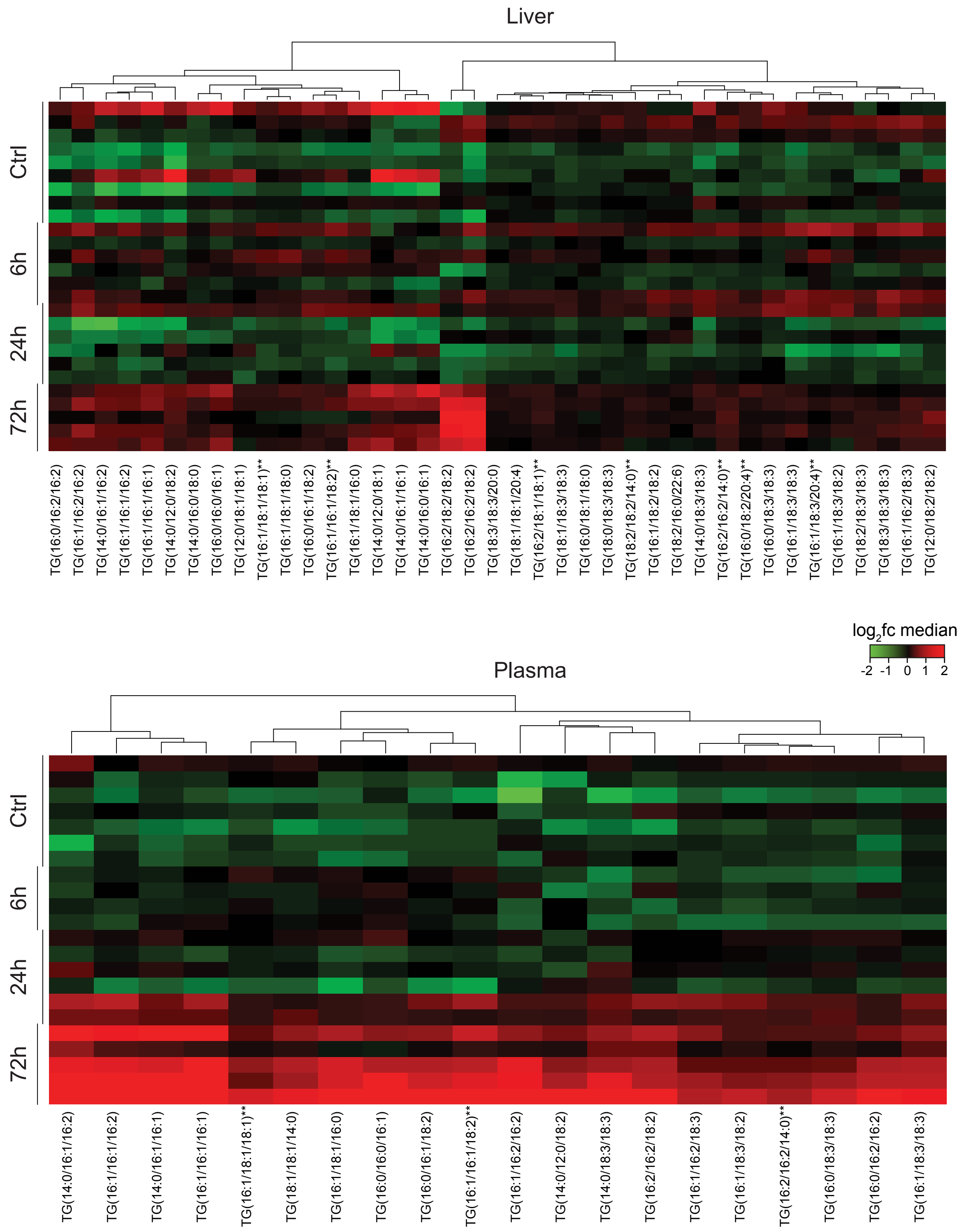
Liver
Ctrl
6h
24h
72h
TG(16:0/16:2/16:2)
TG(16:1/16:2/16:2)
TG(14:0/16:1/16:2)
TG(16:1/16:1/16:2)
TG(16:1/16:1/16:1)
TG(14:0/12:0/18:2)
TG(14:0/16:0/18:0)
TG(16:0/16:0/16:1)
TG(12:0/18:1/18:1)
TG(16:1/18:1/18:1)**
TG(16:1/18:1/18:0)
TG(16:0/16:1/18:2)
TG(16:1/16:1/18:2)**
TG(16:1/18:1/16:0)
TG(14:0/12:0/18:1)
TG(14:0/16:1/16:1)
TG(14:0/16:0/16:1)
TG(16:2/18:2/18:2)
TG(16:2/16:2/18:2)
TG(18:3/18:3/20:0)
TG(18:1/18:1/20:4)
TG(16:2/18:1/18:1)**
TG(18:1/18:3/18:3)
TG(16:0/18:1/18:0)
TG(18:0/18:3/18:3)
TG(18:2/18:2/14:0)**
TG(16:1/18:2/18:2)
TG(18:2/16:0/22:6)
TG(14:0/18:3/18:3)
TG(16:2/16:2/14:0)**
TG(16:0/18:2/20:4)**
TG(16:0/18:3/18:3)
TG(16:1/18:3/18:3)
TG(16:1/18:3/20:4)**
TG(16:1/18:3/18:2)
TG(18:2/18:3/18:3)
TG(18:3/18:3/18:3)
TG(16:1/16:2/18:3)
TG(12:0/18:2/18:2)
log2fc median
-2 -1 0 1 2
Plasma
Ctrl
6h
24h
72h
TG(14:0/16:1/16:2)
TG(16:1/16:1/16:2)
TG(14:0/16:1/16:1)
TG(16:1/16:1/16:1)
TG(16:1/18:1/18:1)**
TG(18:1/18:1/14:0)
TG(16:1/18:1/16:0)
TG(16:0/16:0/16:1)
TG(16:0/16:1/18:2)
TG(16:1/16:1/18:2)**
TG(16:1/16:2/16:2)
TG(14:0/12:0/18:2)
TG(14:0/18:3/18:3)
TG(16:2/16:2/18:2)
TG(16:1/16:2/18:3)
TG(16:1/18:3/18:2)
TG(16:2/16:2/14:0)**
TG(16:0/18:3/18:3)
TG(16:0/16:2/16:2)
TG(16:1/18:3/18:3)

Figure S3

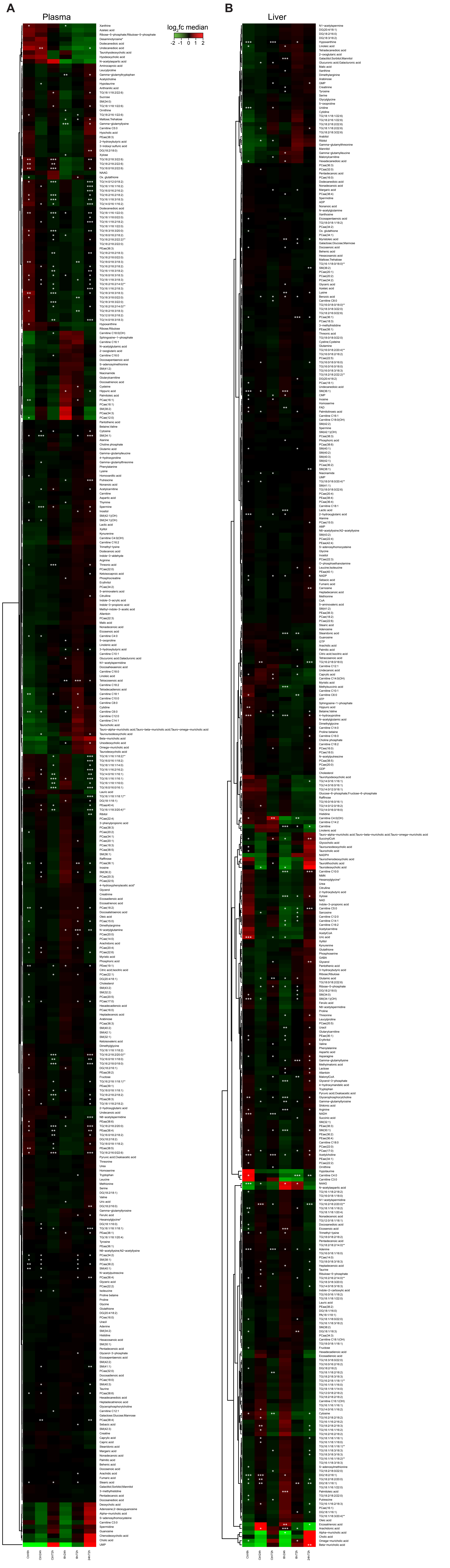

Figure S4

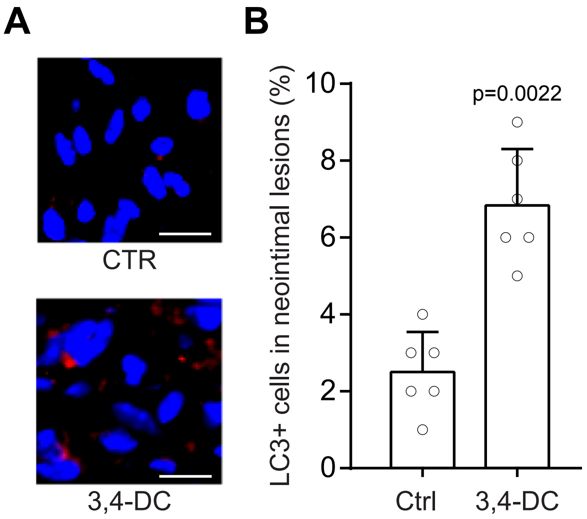
A
B
CTR
3,4-DC
LC3+ cells in neointimal lesions (%)
10
8
6
4
2
0
p=0.0022
Ctrl
3,4-DC

Figure S5

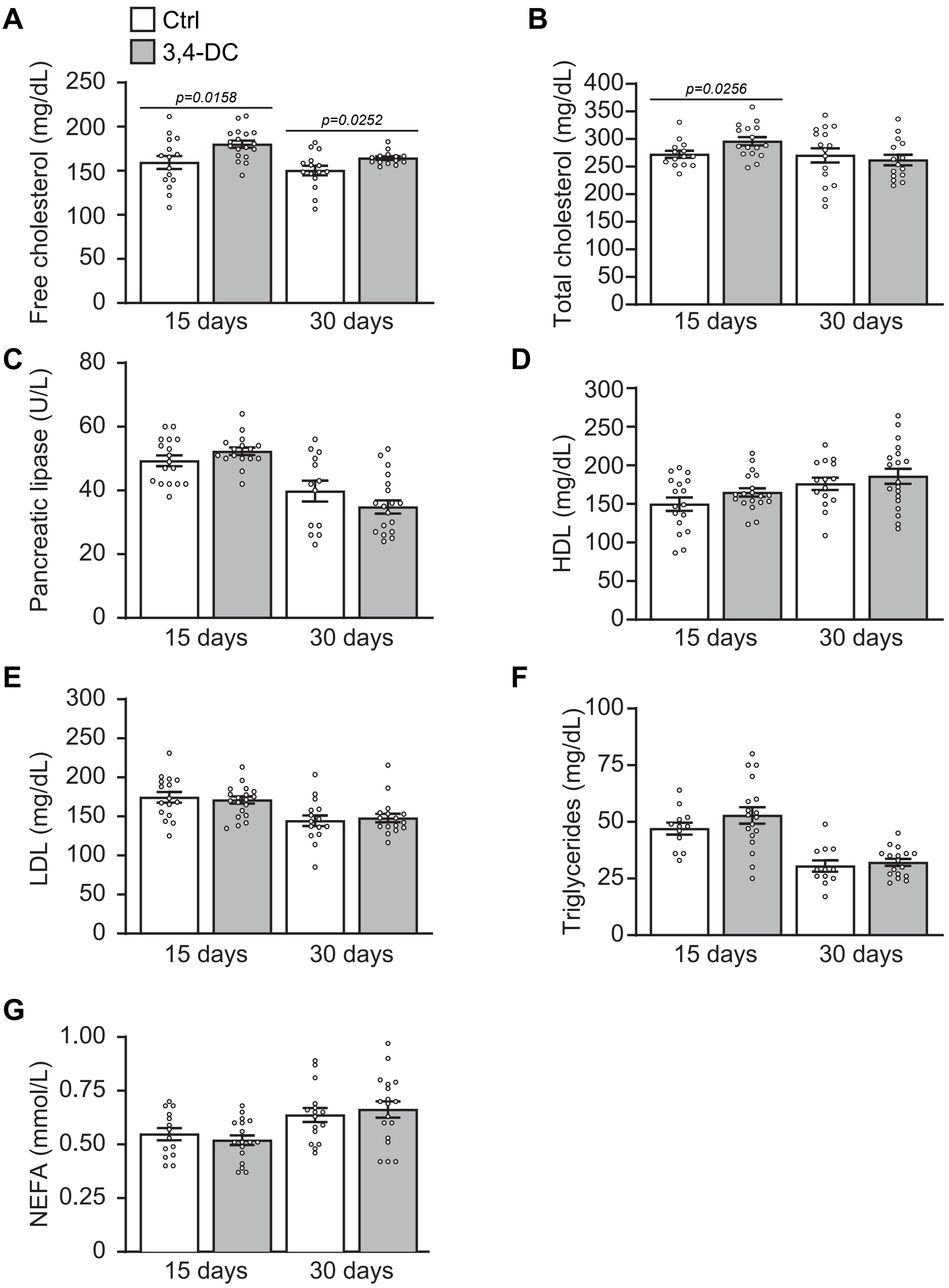
A
Ctrl
3,4-DC
Free cholesterol (mg/dL)
p=0.0158
p=0.0252
15 days
30 days
B
Total cholesterol (mg/dL)
p=0.0256
15 days
30 days
C
Pancreatic lipase (U/L)
15 days
30 days
D
HDL (mg/dL)
15 days
30 days
E
LDL (mg/dL)
15 days
30 days
F
Triglycerides (mg/dL)
15 days
30 days
G
NEFA (mmol/L)
15 days
30 days

Figure S6
